# Prevalence of faecal carriage of extended-spectrum β-lactamase (ESBL)-producing *Escherichia coli* in veterinary hospital staff and students

**DOI:** 10.1136/vetreco-2018-000307

**Published:** 2019-01-07

**Authors:** Alexandra Royden, Emma Ormandy, Gina Pinchbeck, Ben Pascoe, Matthew D Hitchings, Samuel K Sheppard, Nicola J Williams

**Affiliations:** 1 Department of Epidemiology and Population Health, Institute of Infection and Global Health, University of Liverpool, Liverpool, UK; 2 Department of Biology and Biochemistry, The Milner Centre for Evolution, University of Bath, Bath, UK; 3 MRC CLIMB Consortium, University of Bath, Bath, UK; 4 Swansea University Medical School, University of Swansea, Swansea, UK

**Keywords:** antimicrobials, resistance, escherichia coli, epidemiology, zoonoses

## Abstract

Extended-spectrum β-lactamase (ESBL)-producing bacteria causing clinical infections are often also multidrug-resistant (MDR; resistance to ≥3 antimicrobial drug classes), therefore treatment options may be limited. High carriage rates of these potentially zoonotic bacteria have been found in livestock and companion animals. Therefore, people working in veterinary hospitals may be a high-risk population for carriage. This is the first study to determine the prevalence and longitudinal carriage of antimicrobial-resistant (AMR) and ESBL-producing faecal *Escherichia coli* in veterinary hospital staff and students. Prevalence of faecal AMR and ESBL-producing *E coli* was determined in 84 staff members and students in three UK veterinary hospitals. Twenty-seven participants were followed for six weeks to investigate longitudinal carriage. Antimicrobial susceptibility and phenotypic ESBL production were determined and selected isolates were whole genome sequenced. ESBL-producing *E coli* were isolated from five participants (5.95 per cent; 95 per cent CI 0.89 to 11.0 per cent); two participants carried ESBL-producing *E coli* resistant to all antimicrobials tested. Carriage of MDR *E coli* was common (32.1 per cent; 95per cent CI 22.2 to 42.1 per cent) and there was a high prevalence of ciprofloxacin resistance (11.9 per cent; 95 per cent CI 4.98 to 18.8 per cent). ESBL-producing *E coli* were isolated from seven longitudinal participants (25.9 per cent; 95 per cent CI 9.40 to 42.5 per cent); two participants carried ESBL-producing *E coli* for the entire study period. Twenty-six participants (96.3 per cent; 95 per cent CI 89.2 to 100) carried ≥1 MDR *E coli* isolate during the six-week period, with seven participants (25.9 per cent) carrying ≥1 MDR isolate for at least five out of six weeks. The prevalence of faecal ESBL-producing *E coli* in cross-sectional participants is similar to asymptomatic general populations. However, much higher levels of carriage were observed longitudinally in participants. It is vital that veterinary hospitals implement gold-standard biosecurity to prevent transmission of MDR and ESBL-producing bacteria between patients and staff. Healthcare providers should be made aware that people working in veterinary hospitals are a high-risk population for carriage of MDR and ESBL-producing bacteria, and that this poses a risk to the carrier and for transmission of resistance throughout the wider community.

## Introduction

Extended-spectrum β-lactamase (ESBL)-producing *Enterobacteriaceae,* including *Escherichia coli,* present a growing human and veterinary public health risk.^1^ ESBLs are a family of bacterial enzymes, which hydrolyse the β-lactam ring present in β-lactam antimicrobials, conferring resistance to penicillins, second-generation to fourth-generation cephalosporins and monobactams. Of additional concern are AmpC-producing bacteria, which are also resistant to β-lactamase inhibitors.^2^ Horizontal transfer of mobile genetic elements encoding antimicrobial resistance genes in addition to ESBL or plasmid-mediated AmpC β-lactamase genes has led to common multidrug-resistant (MDR) phenotypes.^3^

The emergence of ESBL-producing and AmpC-producing bacteria constitute a major therapeutic burden, contributing to ineffective antimicrobial treatment and presenting economic and public health concerns to healthcare systems worldwide.^1 4^ Thus, it is increasingly important to identify high-risk populations to reduce the threat posed to themselves and other patients. In a study of UK surfers, Leonard *et al* 5 found that 1.5 per cent of a non-surfing, control population were colonised by *E coli*-bearing *bla*_CTX-M_ compared with 6.3 per cent of UK surfers. Intestinal colonisation with ESBL-producing bacteria before hospital admission is associated with increased risk of nosocomial infection.^6^ Patients with infections caused by ESBL-producing bacteria and carriers of these organisms represent a source of resistance and can be responsible for nosocomial and community transmission.^7^ The identification of methicillin-resistant *Staphylococcus aureus* (MRSA) carriers and high-risk groups has been fundamental to successful control policies, such as The Netherlands’ ‘Search and Destroy’ policy.^8^ Similar strategies, including decolonisation regimes, have been proposed for the control of ESBL-producing bacteria in human hospital environments.^6^

Multiple studies have identified antimicrobial-resistant (AMR) faecal *E coli* in small animals,^9–11^ livestock^12–14^ and equids,^15–18^ presenting a direct risk of colonisation to those working with these species, including veterinarians.^19^ However, there is a lack of published estimates of faecal carriage of AMR and ESBL-producing *E coli* by people working in veterinary environments. It is well-documented that carriage of MRSA by veterinarians, pet owners and people occupationally exposed to animals is substantially higher than in healthy, non-veterinary populations, including in the UK.^20^ Thus, it can be hypothesised that carriage of other types of AMR bacteria will be higher among those in veterinary roles and that these individuals provide a reservoir of virulence and resistance genes for transmission throughout other human and animal populations.

This study aimed to investigate the prevalence of faecal carriage of AMR and ESBL-producing *E coli* by staff and students working in three UK veterinary hospitals. Using questionnaires, potential risk factors for faecal carriage of these bacteria were assessed. In addition, a subpopulation of individuals was recruited for a longitudinal study to investigate the length of human faecal carriage of AMR and ESBL-producing *E coli*.

## Methods

### Study population and sampling protocol

Veterinary staff and students were recruited from one equine referral teaching hospital (EH), one small animal referral teaching hospital (SAH) and one farm animal first-opinion and referral teaching hospital (FAH) on the same campus between February and June 2015. Participants were recruited by convenience sampling; the study was advertised to potential participants with posters in each hospital, through email and by word-of-mouth. All participants were aged over 16 years and there were no exclusion criteria.

The prevalence of ESBL-producing bacteria in this healthy human population was estimated to be similar to estimates of other European healthy human populations, approximately 5 per cent. A sample size calculation was performed using the online EpiTools epidemiological calculators^21^ to determine prevalence. From approximately 186 members of staff in the SAH, 61 in the EH, 30 in the FAH and 278 clinical veterinary students, the total number of staff and students within the three hospitals was calculated to be approximately 555. Thus, with an expected prevalence of 5 per cent, precision of 5 per cent and confidence level of 95 per cent, 65 people were required to provide faecal swabs.

Cross-sectional study participants could take part anonymously; provision of a faecal swab and a completed four-page questionnaire regarding potential risk factors for carriage of AMR and ESBL-producing bacteria was considered informed consent (online supplementary material). Participants were asked to provide an email address if they were interested in participating in a longitudinal study where they would be required to provide one faecal swab once a week for a six-week period between May–July 2015. Longitudinal study participants were asked if they had taken antimicrobials, if they or a member of their household had been hospitalised or if they had travelled abroad following participation in the cross-sectional and/or during participation in the longitudinal study.

10.1136/vetreco-2018-000307.supp1 Supplementary data

### Bacterial isolation

Briefly, participants were provided with a sterile cotton swab to collect a small amount of freshly voided faeces. A faecal homogenate was prepared in 1 ml brain-heart-infusion broth with 5 per cent glycerol. Also, 500 µl of homogenate was enriched in 4.5 ml buffered peptone water and incubated aerobically at 37°C for 18–24 hours before culture on three eosin methylene blue agar (EMBA) plates (one plain, one with 1 µg/ml cefotaxime and one with 1 µg/ml ceftazidime). An additional EMBA plate was inoculated with faecal homogenate for confluent bacterial growth and four antimicrobial discs were applied: 10 µg ampicillin, 30 µg amoxicillin-clavulanate, 1 µg ciprofloxacin and 2.5 µg trimethoprim, as previously described.^11 22^ Following aerobic incubation at 37°C for 18–24 hours, three colonies, whose morphology resembled *E coli*, were selected from plain EMBA. One colony growing on each of the cefotaxime and ceftazidime plates and within the zone of inhibition around each antimicrobial disc was selected if present. Thus, up to nine colonies were selected per sample. Antimicrobial discs were obtained from Mast Group (Bootle, UK), media from LabM (Bury, UK) and antibiotic powder from Sigma-Aldrich (Dorset, UK).

### Antimicrobial susceptibility testing

Antimicrobial susceptibility disc diffusion testing was performed according to British Society for Antimicrobial Chemotherapy guidelines^23^ on up to nine colonies per sample. Seven antimicrobial discs were applied to susceptibility plates: 10 µg ampicillin, 30 µg amoxicillin-clavulanate, 30 µg chloramphenicol, 30 µg nalidixic acid, 1 µg ciprofloxacin, 2.5 µg trimethoprim and 30 µg tetracycline. Plates were incubated aerobically at 37°C for 18–24 hours.

### Phenotypic identification of ESBL-producing bacteria

The double disc diffusion test for ESBL production was performed on isolates selected from cephalosporin-containing EMBA plates, as previously described.^11^ ESBL production was confirmed when the zone around the cephalosporin disc was expanded in the presence of the clavulanic acid by a minimum of 5 mm for at least one antimicrobial pair. The AmpC phenotype was suggested when the inhibition zone did not increase with the presence of clavulanic acid, but resistance to at least one cephalosporin was evident.

### Genotypic identification of *E coli* and characterisation of resistance genes by PCR

PCR assays for the *uid*A gene confirmed isolates as *E coli.* 24 All isolates selected from cephalosporin-containing EMBA plates were tested for the presence of predominant ESBL gene variants (*bla*_CTX-M_ 25 (groups 1,^26^ 2^27^ and 9),^28^ *bla*_TEM_, *bla*_SHV_ and *bla*_OXA_ 29). A family-specific multiplex PCR for plasmid-mediated AmpC β-lactamase genes (*bla*_AmpC_) was performed on all isolates selected from cephalosporin-containing EMBA plates and isolates displaying resistance to amoxicillin-clavulanate.^30^

### Whole-genome sequencing (WGS)

Fifty-three *E coli* isolates were selected for WGS; isolates were selected either due to detection of *bla*_CTX-M_, *bla*_TEM_, *bla*_SHV_, *bla*_OXA_ or *bla*_AmpC_ genes by PCR, or for further characterisation of AMR or MDR resistance profiles. Briefly, DNA was extracted from overnight cultures of selected isolates using the QIAamp DNA Mini Kit (Qiagen, Manchester, UK). DNA was then quantified and assessed for purity using a Nanodrop spectrophotometer (ThermoFisher Scientific, Cheshire, UK) before being forwarded to the University of Swansea for WGS. Sequence libraries were prepared using the Nextera XT v2 library preparation kit and sequenced on a MiSeq desktop sequencer (Illumina, San Diego, USA) using MiSeq V3 reagent kits. Genome assemblies of the 300 bp short read pair end data was undertaken using the de novo assembly algorithm SPAdes V.3.3.^31^ Genomes and short read data are archived on the NCBI GenBank and SRA depositories, associated with BioProject PRJNA454281 (https://www.ncbi.nlm.nih.gov/bioproject/PRJNA454281). Individual accession numbers, complete details of the number of contigs and assembled genome size for each of the sequenced isolates are available in online supplementary material. A reference pan-genome approach^32^ with gene-by-gene alignment^33 34^ was implemented using the open source Bacterial Isolate Genome Sequence Database: BIGSdb,^35^ which includes functionality to call MLST profiles defined by the PubMLST database (http://bigsdb.pasteur.fr/ecoli/). MLST types for each isolate were corroborated using the online tool MLST V.1.8^36^ MAFFT software^37^ was used to align gene orthologs and concatenated into contiguous sequence for each isolate genome including gaps. Based on the K12 reference genome (accession: NC_000913.3; 4319 loci) a core genome alignment (genes present in 90 per cent or more isolates) was constructed (3,344,351 bp; 3473 loci) and a heuristic maximum-likelihood tree generated using FastTree2 (V.2.1.0).^38^ Core genome genealogies and meta-data were visualised using Microreact^39^ and shared: https://microreact.org/project/RoydenVetEcoli.

10.1136/vetreco-2018-000307.supp2 Supplementary data

The online tools ResFinder V.3.0^40^ and VirulenceFinder V.1.5^41^ were used to determine carriage of antimicrobial resistance genes and virulence genes. PlasmidFinder V.1.3^42^ was used to assess for the presence of plasmid replicons and pMLST V.1.4^42^ and the plasmid MLST website (https://pubmlst.org/plasmid/)^35^ allocated Inc replicon sequence types (RSTs) for isolates where PlasmidFinder identified one or more incompatibility groups for which schemes are available (IncF, IncHI1, IncHI2, IncN and IncI1). In silico serotyping of isolates was undertaken using SerotypeFinder V.1.1^43^ in order to assess the O and H serogroups of each isolate.

#### Statistical analysis

Twenty-four independent, binomial and categorical predictor variables were created from 84 cross-sectional study participant questionnaires. Outcome data for AMR *E coli* were collapsed to the sample level. Therefore, a sample with at least one resistant faecal *E coli* isolate was classed as resistant for analysis. Fourteen binomial antimicrobial resistance outcomes were considered as response variables: (i) ampicillin resistance, (ii) amoxicillin-clavulanate resistance, (iii) nalidixic acid resistance, (iv) ciprofloxacin resistance, (v) chloramphenicol resistance, (vi) trimethoprim resistance, (vii) tetracycline resistance (viii) β-lactam resistance (ix) quinolone resistance, (x) third-generation cephalosporin resistance, (xi) resistance to ≥1 tested antimicrobial, (xii) MDR=resistance to≥3 tested antimicrobials, (xiii) resistance to all five tested antimicrobial classes (β-lactams, quinolones, chloramphenicol, trimethoprim and tetracycline) and (xiv) sample containing ESBL-producing *E coli*. Univariable logistic regression models analysed the association between all independent predictor variables and resistance outcomes (online supplementary material). Variables were tested in multivariable models if the likelihood-ratio test P value<0.25. Collinearity between explanatory variables was assessed using Pearson’s chi-square test for independence, or if n<5, Fisher’s exact test. For variables with a significant association (P<0.05) only variables with the lowest P value were considered for inclusion in the multivariable models. Final models were constructed by manual backwards stepwise procedures where variables with a likelihood-ratio test P-value<0.05 were retained. The fit of the model was then tested using graphical residual analysis.

10.1136/vetreco-2018-000307.supp3 Supplementary data

Pearson’s chi-square test for independence, or if n<5, Fisher’s exact test, was performed to determine any statistically significant differences in AMR data between the three veterinary teaching hospitals. All statistical tests were performed using R (R V.3.2.0 for Mac OS X).^44^

## Results

### Cross-sectional study

#### Study population

From a population of approximately 555 potential study participants, a total of 84 participants were recruited, and their occupations and workplaces are shown in table 1.

**Table 1:** Occupations and workplaces of 84 cross-sectional study participants

	FAH	EH	SAH	Total
Veterinary surgeon	13	13	23	49
Veterinary nurse	0	2	3	5
Veterinary student	15	5	4	24
Auxiliary staff	2	0	1	3
Administrative or other role	2	0	1	3
Total	32	20	32	84

EH, equine hospital; FAH, farm animal hospital; SAH, small animal hospital.

#### Phenotypic antimicrobial resistance

*E coli* were isolated from 78 samples (92.9 per cent; 95 per cent CI: 87.3 to 98.4 per cent). Of these, 27 (32.1 per cent; 95 per cent CI 22.2 to 42.1 per cent) samples contained at least one MDR (resistance to ≥3 antimicrobial drug classes) *E coli* and 6 (7.14 per cent; 95 per cent CI 1.64 to 12.7 per cent) contained at least one *E coli* isolate resistant to all five antimicrobial classes tested. There was a notably high prevalence of resistance to ciprofloxacin (11.9 per cent; 95 per cent CI 4.98 to 18.8 per cent). The percentage of samples containing at least one *E coli* isolate resistant to each of the tested antimicrobials is shown in table 2. ESBL-producing *E coli* were isolated from five samples (5.95 per cent: 95 per cent CI 0.89 to 11.0 per cent); two of these samples (E38 and S57) contained MDR ESBL-producing *E coli*, resistant to all antimicrobials tested (table 3). Comparisons between the three hospitals revealed a significantly higher prevalence of trimethoprim-resistant *E coli* in the FAH compared with the SAH (Χ^2^=7.09, P=0.008).

**Table 2:** The percentage of samples from 84 participants (95% CI; N) containing at least one faecal *E coli* isolate resistant to the tested antimicrobials; overall and stratified by hospital

Resistance	All participants	FAH	EH	SAH
AMP	53.6 (42.9 to 64.2; 45)	56.3 (39.1 to 73.4; 18)	55.0 (33.2 to 76.8; 11)	50.0 (32.7 to 67.3; 16)
AMC	10.7 (4.10 to 17.3; 9)	15.6 (3.04 to 28.2; 5)	5.0 (0 to 14.6; 1)	9.38 (0 to 19.5; 3)
NAL	25.0 (15.7 to 34.3; 21)	28.1 (12.5 to 43.7; 9)	25.0 (6.02 to 44.0; 5)	21.9 (7.55 to 36.2; 7)
CIP	11.9 (4.98 to 18.8; 10)	15.6 (3.04 to 28.2; 5)	10.0 (0 to 23.1; 2)	9.38 (0 to 19.5; 3)
CHL	16.7 (8.70 to 24.6; 14)	15.6 (3.04 to 28.2; 5)	20.0 (2.47 to 37.5; 4)	15.6 (3.04 to 28.2; 5)
TMP	34.5 (24.4 to 44.7; 29)	50.0 (32.7 to 67.3; 16)	40.0 (18.5 to 61.5; 8)	15.6 (3.04 to 28.2; 5)
TET	39.3 (28.8 to 49.7; 33)	46.9 (29.6 to 64.2; 15)	40.0 (18.5 to 61.5; 8)	31.3 (15.2 to 47.3; 10)
BLM	53.6 (42.9 to 64.2; 45)	56.3 (39.1 to 73.4, 18)	55.0 (33.2 to 76.8; 11)	50 (32.7 to 67.3; 16)
QNL	25.0 (15.7 to 34.3; 21)	28.1 (12.5 to 43.7; 9)	25.0 (6.02 to 44.0; 5)	21.9 (7.55 to 36.2; 7)
3GCR	10.7 (4.10 to 17.3; 9)	9.38 (0 to 19.5; 3)	5.0 (0 to 14.6; 1)	15.6 (3.04 to 28.2; 5)
AMR	60.7 (50.3 to 71.2, 51)	59.4 (42.4 to 76.4; 19)	65.0 (44.1 to 85.9; 13)	59.4 (42.4 to 76.4; 19)
MDR	32.1 (22.2 to 42.1; 27)	40.6 (23.6 to 57.6, 13)	35.0 (14.1 to 55.9; 7)	21.9 (7.55 to 36.2; 7)
ALL	7.14 (1.64 to 12.7; 6)	12.5 (1.04 to 24.0; 4)	5.0 (0 to 14.6; 1)	3.13 (0 to 9.15; 1)
ESBL	5.95 (0.892 to 11.0; 5)	3.13 (0 to 9.15; 1)	5.0 (0 to 14.6; 1)	9.38 (0 to 19.5; 3)

ALL, resistance to all five tested antimicrobial classes (β-lactams, quinolones, chloramphenicol, trimethoprim and tetracycline); AMC, amoxicillin clavulanate resistance; AMP, ampicillin resistance; AMR, resistance to≥1 tested antimicrobial; BLM, β-lactam resistance; CHL, chloramphenicol resistance; CIP, ciprofloxacin resistance; EH, equine hospital; ESBL, sample contained ESBL-producing *E coli*.; FAH, farm animal hospital; MDR, resistance to≥3 tested antimicrobials; NAL, nalidixic acid resistance; QNL, quinolone resistance; SAH, small animal hospital; TET, tetracycline resistance; TMP, trimethoprim resistance; 3GCR, third-generation cephalosporin resistance

**Table 3:** Antimicrobial resistance phenotype and characterisation by whole genome sequencing (WGS) of 29 *E coli* isolates from 84 cross-sectional study participants, ordered by MLST sequence type

Isolate (participant†)	Phenotype	MDR	ESBL (3GCR)	ST	Serotype	Plasmid type	Antimicrobial Resistance Genes	Virulence Genes
101 (F18)	AmpChlNalCipTmpTet	Y	–-(–)	10 (CC-10)	O9:H9	IncF RST F67:A6:B38	*aadA1, bla*_TEM-1A_, *dfrA1, floR, strA, strB, sul2, tet(A), tet(B)*	*astA, gad, mchB, mchC, mchF*
380 (S74)	AmpNalCipTmpTet	Y	–	10 (CC-10)	H4	Col(MP18)*; Col(BS512); Col156*; IncI1(ST-3);	*bla*_TEM-1B_, *dfrA1, QnrB19, sul2, tet(A)*	*gad*
386 (S76)	AmpNalTmpTet	Y	–	13 (CC-13)	H8	Col(MP18)*; IncB/O/K/Z*; IncF RST C3*:A-:B1, IncQ1;	*aadA1, bla*_TEM-1B_, *dfrA1, strA, strB, sul1, sul2, tet(A)*	*gad, iha, iss, lpfA*
59 (F11)	TmpTet	N	– (–)	58	O9:H25	Col156; IncF RST F4:A-:B1; IncQ1	*aph(3')-Ia, bla*_TEM-1B_, *dfrA5, strA, strB, sul2, tet(A)*	*air, capU, gad, iha, ireA, iroN, iss, lpfA, mchF, mcmA, senB*
36 (F04)	AmpTmpTet	Y	– (–)	59 (CC-59)	H7	Col156*; IncF RST F4:A-:B10	*bla*_TEM-1C_, *dfrA17, tet(A)*	*air, capU, eilA, gad, iha, lpfA, sat, senB*
161 (F30)	AmpTmpTet	Y	– (–)	69 (CC-69)	H1	Col156*, IncF RST F87*:A4:B10, IncI1(ST-242)*	*aadA5, bla*_TEM-1B_, *dfrA17, sul1*	*air, gad, iss, lpfA, senB*
181 (E33)	AmpNalTmpTet	Y	– (–)	69 (CC-69)	O15:H18	Col156*; IncB/O/K/Z; IncF RST F52:A6*:B48, IncI1 Incomplete ST; IncQ1;	*bla*_TEM-1B_, *dfrA7, strA, strB, sul1, sul2, tet(A)*	*air, celb, eilA, gad, iha, ireA, iroN, iss, lpfA*
121 (F22)	AmpChlTet	Y	– (–)	88 (CC-23)	O9:H19	IncF RST F2:A-:B-, IncI1 Unknown ST, IncI2*	*aadA1, catA1, strA, strB, tet(B)*	*gad, iss, lpfA, mcmA*
299 (S59)	Amp	N	Y (Y)	127	O6:H31	Col(BS512); Col(MG828)*; Col156*; IncB/O/K/Z; IncF RST F29:A-:B10, IncI1 Incomplete ST; IncX1*;	*aadA5, aadA15, bla*_CTX-M-14_, *bla*_TEM-1A_, *cml, dfrA12, strB, sul1, tet(B)*	*celb, gad, ireA, iss, lpfA, senB, vat*
350 (S68)	AmpNalCip	N	Y (Y)	131	O25:H4	Col(BS512); Col156*; IncF RST F1:A2:B20	*bla*_CTX-M-27_	*gad, iha, iss, sat, senB*
151 (S27)	AmpTmpTet	Y	– (–)	141	H6	IncI1(ST223*); IncX1*; p0111*;	*aac(3)-IIa, aadA1, aph(3')-Ia, bla*_TEM-1B_, *dfrA1, strA, strB, sul2, tet(A)*	*astA, gad, iroN, iss, mchB, mchC, mchF, mcmA, vat*
229 (E42)	AmpChlTmpTet	Y	– (–)	141	O50/O2:H6	IncF RST F40:A-:B-, IncQ1;	*aac(3)-IId, aac(6')Ib-cr, aacA4, ARR-3, bla*_TEM-1B_, *catA1, catA2, dfrA12, mph(E), msr(E), strA, strB, sul1, sul2, tet(A)*	*cba, cma, gad, iroN, iss, mchB, mchC, mchF, mcmA, vat*
200 (E38)	AmpChlNalCipTmpTet	Y	Y (Y)	405 (CC-405)	H6	IncF RST F1:A1:B49, IncQ1;	*aac(3)-IId, aac(6')Ib-cr, aadA5, bla*_CTX-M-15_, *bla*_OXA-1_, *bla*_TEM-1B_, *catB4, dfrA17, mph(A), strA, strB, sul1, sul2, tet(B)*	*air, gad*
288 (S57)	AmpAmcChlNalCipTmpTet	Y	Y (Y)	405 (CC-405)	O102:H6	Col(BS512); Col156*; IncF RST F87*:A4:B1, p0111*;	*aac(6')Ib-cr, aadA5, bla*_CTX-M-15_, *bla*_OXA-1_, *catB4, dfrA17, mph(A), strB, sul1, tet(B)*	*air, celb, eilA, gad, iha, ireA, iroN, iss, lpfA*
254 (E49)	AmpAmcChlNalCipTmp	Y	– (–)	410 (CC-23)	H9	IncI1(ST-26*)	*aadA1, bla*_OXA-1_, *bla*_TEM-33_, *floR, sul1, sul2*	*gad, lpfA*
258 (E49)	AmpAmcNalCip	N	– (–)	410 (CC-23)	H9	IncI1(ST-26*)	*aadA1, bla*_OXA-1_, *bla*_TEM-33_, *strB, sul1*	*gad, lpfA*
12 (F03)	AmpNalCip	N	Y (Y)	648	H6	IncF RST F4:A-:B52, p0111*	*aac(3)-IIa, aac(6')Ib-cr, bla*_CTX-M-15_, *bla*_OXA-1_, *bla*_TEM-1B_, *catB4*	*air, gad, lpfA, nfaE, sat*
17 (F03)	AmpNalCipTet	Y	– (–)	648	H6	IncF RST F4:A-:B52, p0111*	*aac(3)-IIa, aac(6')Ib-cr, bla*_CTX-M-15_, *bla*_OXA-1_, *bla*_TEM-1B_, *catB4*	*air, eilA, gad, lpfA, nfaE, sat*
91 (F16)	AmpChlNalCipTmpTet	Y	– (–)	744	O89:H10	IncHI1(ST6*), IncQ1	*aadA5, aph(3')-Ia, bla*_TEM-1B_, *catA1, dfrA17, mph(A), strA, strB, sul1, sul2, tet(B)*	*gad, lpfA, pic, senB, vat*
175 (F32)	AmpAmcTmpTet	Y	– (–)	778	H18	IncF RST F1:A1*:B1, IncQ1	*bla*_TEM-1B_, *dfrA14, dfrA8, mph(A), strA, strB, sul2, tet(A)*	*aap, astA, gad, iss, nfaE*
331 (S64)	AmpAmc	N	N (Y)	963	H18	Col(BS512)*; Col156*; IncF RST F29:A-:B10	*bla*_CMY-2_	*air, gad, senB*
05 (F01)	AmpAmcChlNalTmpTet	Y	– (–)	1861	O16:H5	Col(BS512), IncF RST F31*:A-:B10*, IncI1 Incomplete ST	*dfrA17*	*gad, lpfA, pic, senB, vat*
199 (E37)	AmpNalTet	Y	– (–)	2076	O17/44:H18	IncF RST F77:A-:B12*	*bla*_TEM-1B_	*air, capU, celb, eilA, gad, lpfA*
266 (F51)	AmpAmcNalCip	N	N (Y)	3944	O8:H2	IncI1(ST-12); IncY*	*bla*_CMY-2_	*gad*
267 (F51)	AmpAmcChlNalCipTmp	Y	N (Y)	3944	O8:H2	IncI1(ST-12); IncX1*; IncY*	*aadA1, aadA2, bla*_CMY-2_, *cmlA1, dfrA12, sul3*	*gad*
73 (F13)	AmpAmcNalTmpTet	Y	– (–)	6899	H18	IncF RST F40:A-:B-, IncN(ST3)	*bla*_TEM-1B_, *QnrS1, strA, strB, sul2, tet(A)*	*air, eilA, gad, iss, lpfA*
123 (F22)	AmpAmcChlNalCipTmpTet	Y	– (–)	Unknown	O9	IncF RST F-:A-:B38	*aadA1, dfrA1, strB, sul1, sul2, tet(B)*	*astA, gad, iss*
131 (E24)	AmpChlTet	Y	– (–)	Unknown	Unknown	IncHI1(ST11*)	*bla*_SHV-40_, *fosA*	None Detected
411 (E79)	Amp	N	N (N)	Unknown	Unknown	IncF RST F-:A-:B50*	None Detected	*gad, iss*

*Allelic match of <100%.

† Letter in front of participant’s ID number indicates workplace (E = equine hospital, S =small animal hospital, F =farm animal hospital).

–, not tested; 3GCR, resistance to at least one third-generation cephalosporin on double disc diffusion testing; Amc, amoxicillin clavulanate resistance; Amp, ampicillin resistance; CC, clonal complex; Chl, chloramphenicol resistance; Cip, ciprofloxacin resistance; ESBL, phenotypic ESBL-producer on double disc diffusion testing; MDR, multidrug resistance; n, no; Nal, nalidixic acid resistance; ST, sequence type; Tmp, trimethoprim resistance; Tet, tetracycline resistance; Y, yes.

A total of 151 unique *E coli* isolates were identified from the antimicrobial resistance profiles of all *E coli* isolates from 84 samples (online supplementary material). From a large diversity of resistance profiles, the most common resistance profile was to ampicillin, followed by ampicillin-trimethoprim-tetracycline. Nineteen participants were found to be carrying more than one unique *E coli* isolate with different AMR profiles.

#### Characterisation of resistance genes and WGS

In total, 29 unique *E coli* isolates from 25 cross-sectional study participants were selected for WGS either due to detection of *bla*_CTX-M_, *bla*_TEM_, *bla*_SHV_, *bla*_OXA_ or *bla*_AmpC_ genes by PCR, or for further characterisation of their AMR or MDR resistance profiles (table 3). Examination of isolates from the five samples carrying phenotypic ESBL-producing *E coli* revealed *bla*_CTX-M-15_ in four isolates, and *bla*_CTX-M-14_ and *bla*_CTX-M-27_ individually in two isolates. Through WGS, three additional *E coli* isolates were confirmed as putative ESBL producers, carrying *bla*_TEM-33_ or *bla*_SHV-40_ genes, and three isolates were revealed to carry *bla*_CMY-2_.

Examination of the 29 isolates revealed genes conferring resistance to ten antimicrobial classes; the most common being the aminoglycoside resistance genes, *strA* (n=11) and *strB* (n=15), the sulphonamide resistance genes, *sul1* (n=11) and *sul2* (n=13), and the tetracycline resistance gene, *tet(A)* (n=10). Aside from the 10 *bla* genes identified, of which 6 encode ESBL or AmpC enzymes, 36 other resistance genes were recognised; of which 17 were only identified once. Specific examination of further resistance genes carried by the 12 isolates harbouring the six plasmid-mediated *bla* genes encoding ESBL or AmpC enzymes identified 21 other class-specific resistance genes, with individual isolates carrying up to 11 resistance genes in addition to any *bla* genes. As can be seen in table 3, WGS highlighted the presence of resistance genes for which the isolates demonstrated phenotypic resistance on antimicrobial susceptibility testing.

In silico plasmid replicon typing revealed plasmids belonging to 10 replicon groups, with all isolates carrying at least 1 replicon type and one isolate 6 replicon types. Eighteen different IncF RSTs were identified. Two isolates (73 and 229) were identified as carrying the same plasmid replicon type IncF RST F40:A-:B- and both carried *bla*_TEM-1B_, *strA, strB, sull2, tet(A), gad* and *iss* genes. Additionally, Isolates 299 and 331, isolated from two participants working in the SAH, were both carrying a IncF RST F29:A-:B10 plasmid and both carried the *gad* and *senB* (plasmid-encoded enterotoxin *tieB*) genes. From the 11 isolates harbouring an IncI1 replicon, 5 IncI1 groups were identified. In silico serotyping designated 21 isolates an O serogroup and all 29 isolates a H serogroup. In total, 12 O antigens and 14 H antigens were identified.

**Figure 1:**
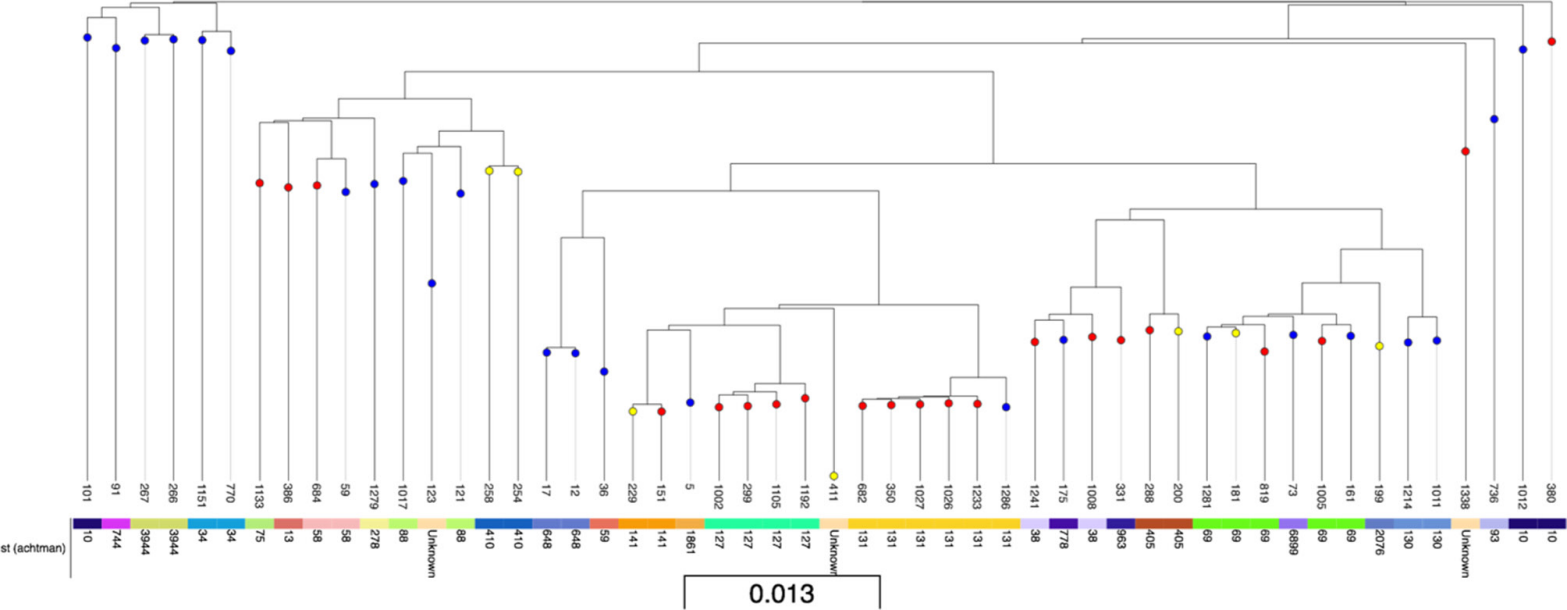
Heuristic maximum-likelihood tree of 53 isolates generated using FastTree2 (V.2.1.0).^38^ Core genome genealogies and meta-data were visualised using Microreact.^39^ Further visualisation with extensive isolate metadata is available at https://microreact.org/project/RoydenVetEcoli. Taxa are labelled with isolate number. Colour of node represents workplace of participant isolate was isolated from: yellow = equine hospital, blue = farm animal hospital, red = small animal hospital. Metadata represents MLST type (Achtman) of isolates. The scale bar (0.013) indicates the number of substitutions per site

WGS revealed a range of 19 different MLST types in the 29 isolates; 15 STs were specific to one participant and 4 STs (ST10, ST69, ST141 and ST405) occurred in more than one participant. The 14 isolates confirmed as carrying *bla* genes encoding ESBL or AmpC enzymes belonged to eight different STs. ST405 was identified in two participants carrying MDR phenotypic ESBL-producing isolates with the *bla*_CTX-M-15_ gene. The heuristic maximum-likelihood phylogenetic tree of 53 isolates (figure 1) indicates that there is very little relationship between the workplace of the participant the isolate was collected from and the isolates’ relatedness. The ESBL-producing isolates from different participants were not clustered closely together. Further visualisation of the core genome genealogies with extensive isolate metadata is available using Microreact^39^ at https://microreact.org/project/RoydenVetEcoli.

#### Risk factors for carriage of AMR *E coli*

Univariable logistic regression analysis is available in online supplementary material. One significant multivariable logistic regression model was constructed; workplace, direct contact with animal faeces at work and hospitalisation in the last six months were independently associated with carriage of trimethoprim-resistant *E coli* (P<0.001) (table 4).

**Table 4:** Final multivariable logistic regression model for carriage of trimethoprim-resistant (Tmp-R) *E coli* in 84 cross-sectional study participants

Resistance outcome	Covariates	Tmp-R positive (n=29)	Tmp-R negative (n=55)	B	SE (b)	Adjusted OR	95% CI	P value*
Trimethoprim-resistance (P=0.00109†)	Farm animal hospital (reference category)	16	16	−	−	−	−	0.00324
	Equine hospital	8	12	−0.29	0.62	0.75	0.22 to 2.51	0.639
	Small animal hospital	5	27	−2.13	0.74	0.12	0.02 to 0.45	0.00389
	Direct contact with animal faeces at work	24	51	−2.19	0.99	0.11	0.01 to 0.72	0.0208
	Hospitalisation in the last six months	4	1	2.30	1.26	9.97	1.14 to 235.15	0.0371

If not specified, the reference category is the absence of the risk factor.

*P value from Wald test.

†P value from likelihood-ratio test statistic.

### Longitudinal study

Twenty-seven cross-sectional study participants were recruited for the longitudinal study; 24 veterinary surgeons, 1 veterinary student, 1 veterinary nurse and 1 auxiliary staff member. Eleven longitudinal participants were from the FAH, eight from the EH, seven from the SAH and one veterinary student on hospital rotations.

**Figure 2:**
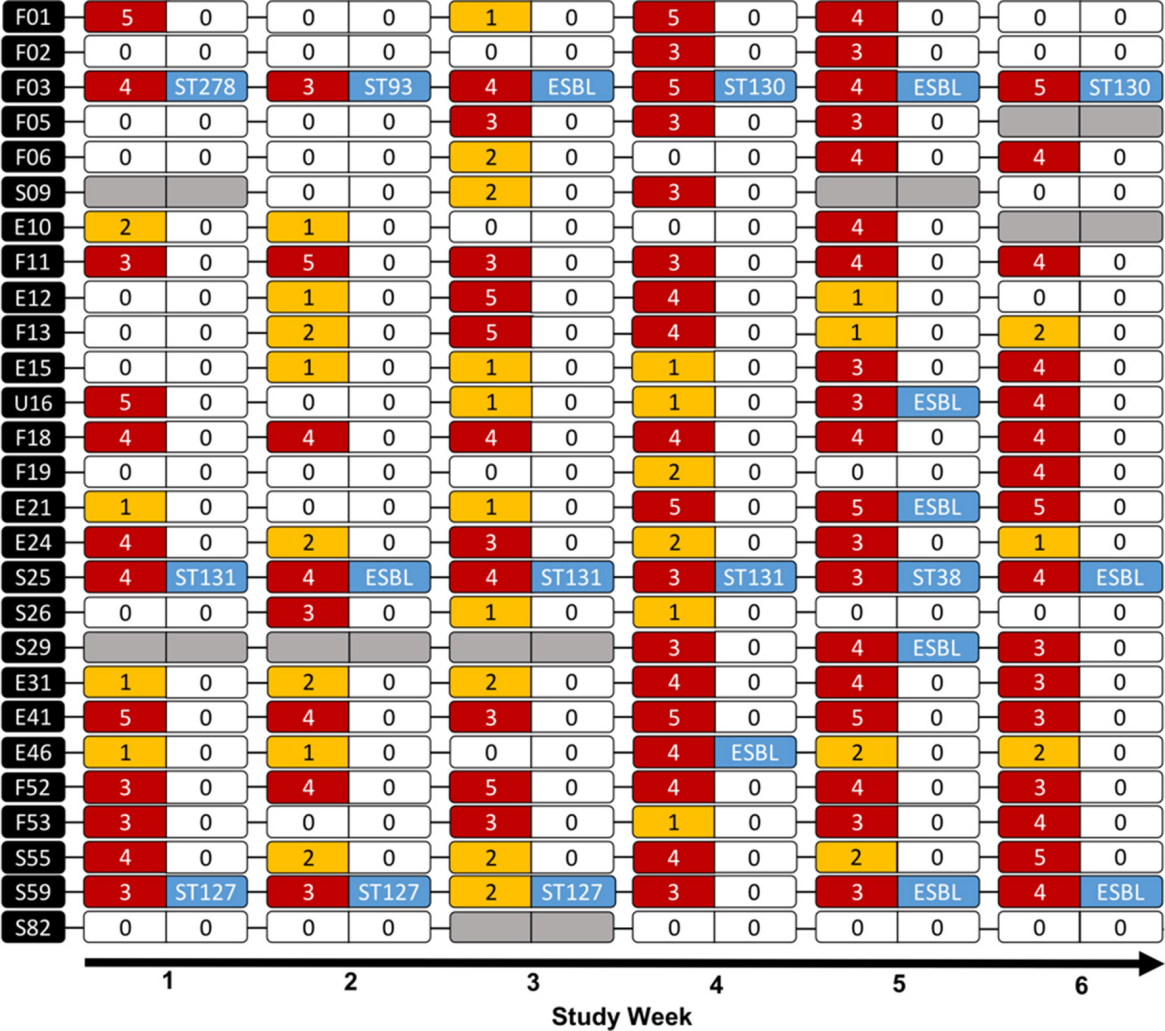
Timeline of results for 27 longitudinal study participants. Each row of six capsules represents the faecal carriage results of one participant. The black capsule at the start of each row is the participant’s ID number. The letter in front of each participant’s ID number indicates workplace (E=equine hospital, S=small animal hospital, F=farm animal hospital, U=undergraduate veterinary student). Each capsule of two halves represents the results for one week: the left side represents isolation by culture of antimicrobial-resistant (AMR) *E coli*, with the number of antimicrobial classes *E coli* in the sample demonstrated resistance to, the right side represents identification of Extended-spectrum β-lactamase (ESBL)-producing *E coli* and ST from whole genome sequencing (WGS) if available. Grey/blank capsules indicate no *E coli* isolated from that week’s sample.

Twenty-six participants (96.3 per cent; 95 per cent CI 89.2 to 100 per cent) were found to carry at least one MDR faecal *E coli* during the six-week period, with 16 participants (59.3 per cent) carrying at least one AMR *E coli* isolate and 7 participants (25.9 per cent) carrying at least one MDR *E coli* isolate for at least five out of six weeks (figure 2). Phenotypic ESBL-producing *E coli* were isolated from seven participants (25.9 per cent; 95 per cent CI 9.40 to 42.5 per cent) during the study period; with three participants (F03, S25 and S59) demonstrating persistent carriage in at least five out of six samples. Further characterisation by PCR and WGS revealed weekly similarities and variations in strains and resistance phenotypes between isolates from these three participants (table 5).

**Table 5:** *E coli* isolates with *bla*_CTX-M_, *bla*_TEM_, *bla*_SHV_, *bla*_OXA_ or *bla*_AmpC_ genes as determined by PCR assay^25–30^ and/or characterised by whole-genome sequencing (WGS) from 16 (n=27) longitudinal study participants over a six-week study period

Participant* (occupation)	Week	Isolate	Resistance profile	MDR	Phenotypic ESBL (3GCR)	Resistance genes by PCR assay	ST (CC)	Serotype	Plasmid type	Antimicrobial resistance genes	Virulence genes
F01 (Vet)	1	416	AmpNalCipChlTmpTet	Y	N (–)	*bla*_AmpC_ (CIT)	–	–	–	–	–
F03 (Vet)	1	1279	AmpTmp	N	Y (Y)	*bla*_CTX-M_ Grp 1	278 (CC-278)	O131:H4	ColpVC*, IncB/O/K/Z*, IncF RST F17:A-:B5, IncI1 Incomplete ST	*aadA5, bla*_CTX-M-15_, *dfrA17, mph(A), sul1*	*aap, aaiC, aatA, aggR, capU, gad, iha, iss, lpfA, mchB, mchC, mchF, ORF4, pic, sat, sepA*
		1281	AmpTmpTet	Y	– (–)	*–*	69 (CC-69)	O15:H18	IncF RST F35:A6:B48, IncQ1	*bla*_TEM-1B_, *dfrA7, strA, strB, sul1, sul2, tet(A)*	*air, eilA, gad, iha, iroN, iss, lpfA*
		1286	AmpNalCipTmpTet	Y	– (–)	–	131	O16:H5	Col156*, IncF RST F1:A1:B66	*bla*_CTX-M-15_, *dfrA14, mph(A), tet(B)*	*gad, iha, sat, senB*
	2	735	AmpTmp	N	Y (Y)	*bla*_CTX-M_ Grp 1	–	–	*–*	–	*–*
		736	Amp	N	Y (Y)	*bla*_CTX-M_ Grp 1, *bla*_TEM_	93 (CC-168)	O88:H38	Col(BS512), IncB/O/K/Z, IncF RST F58:A-:B-	*aadA1, bla*_CTX-M-15_, *bla*_TEM-1B_, *QnrS1*	*aap, astA, gad, iss*
	3	769	AmpTmp	N	Y (Y)	*bla*_CTX-M_ Grp 1	–	–	*–*	–	*–*
		768	Amp	N	Y (Y)	*bla*_CTX-M_ Grp 1, *bla*_TEM_	–	–	*–*	–	*–*
		770	AmpNalTmp	Y	– (–)	*–*	34 (CC-10)	O62:H30	Col(BS512), Col156*, IncB/O/K/Z, IncF RST F36*:A-:B-, IncI1 Incomplete ST	*bla*_TEM-1B_, *dfrA12, dfrA14, strA, strB, sul2, sul3, tet(A)*	*aap, capU, cba, gad, iha, iss, mchB, mchC, mchF*
	4	1010	AmpTmp	N	Y (Y)	*bla*_CTX-M_ Grp 1	–	–	*–*	–	*–*
		1011	AmpChlTmp	Y	Y (Y)	*bla*_CTX-M_ Grp 1, *bla*_TEM_	130 (CC-31)	O15:H12	IncF RST F1:A6*:B33, IncQ1	*aac(3)-IId, aadA2, bla*_CTX-M-15_, *bla*_TEM-1B_, *catA1, dfrA12, mph(A), strA, strB, sul1, sul2, tet(A)*	*aap, aaiC, aar, agg3A, agg3B, agg3D, aggR, capU, eilA, gad, iha, iroN, iss, lpfA, nfaE, ORF4, pic*
		1012	AmpTmpTet	Y	– (–)	*–*	10 (CC-10)	O2:H48	Col(BS512), Col(MG828)*, Col156*, IncF RST F98:A-:B65	*bla*_TEM-1B_, *strA, strB, sul2, tet(A)*	*gad, iha, senB, sigA*
		1017	AmpAmcChlNalCipTmpTet	Y	– (–)	*–*	88 (CC-23)	O25:H17	Col156*, IncF RST F67:A6:B38	*aadA1, aph(3')-Ic, dfrA1, floR, strB, sul1, sul2, tet(B)*	*astA, gad, iha, iss, lpfA, mcmA, senB*
	5	1149	AmpChlTmp	Y	Y (Y)	*bla*_CTX-M_ Grp 1, *bla*_TEM_	–	–	*–*	–	*–*
		1151	AmpNalTmp	Y	– (–)	*–*	34 (CC-10)	H30	Col(BS512), IncB/O/K/Z, IncF RST F2:A-:B-	*bla*_TEM-1C_, *dfrA14, strA, strB, sul2*	*aap, capU, cba, gad, iha, mchB, mchC, mchF, sat*
	6	1214	AmpChlTmp	Y	Y (Y)	*bla*_CTX-M_ Grp 1, *bla*_TEM_	130 (CC-31)	O17/O44:H1	IncF RST F1:A-:B33	*aac(3)-IId, aadA2, bla*_CTX-M-15_, *bla*_TEM-1B_, *catA1, dfrA12, mph(A), sul1*	*aap, aaiC, aar, agg3B, agg3C, agg3D, air, capU, eilA, gad, iha, lpfA, nfaE, ORF3, ORF4, pic*
F06 (Vet)	3	662	AmcTet	N	N (Y)	*bla*_AmpC_ (ACC)	–	–	*–*	–	*–*
E10 (Vet)	1	442	AmpAmc	N	N (Y)	*bla*_AmpC_ (CIT)	–	–	*–*	–	*–*
F13 (Vet)	2	615	AmpAmcTet	N	N (Y)	*bla*_TEM_, *bla*_OXA_	–	–	*–*	–	*–*
E15 (Vet)	2	479	None	N	N (N)	*bla*_OXA_	–	–	*–*	–	*–*
	4	701	Amp	N	N (-)	*bla*_AmpC_ (ACC)	–	–	*–*	–	*–*
U16 (UVS)	5	871	Chl	N	Y (Y)	*bla*_CTX-M_ Grp 9	–	–	*–*	–	*–*
E21 (Vet)	5	1018	AmpChlTet	Y	Y (Y)	*bla*_CTX-M_ Grp 1	–	–	*–*	–	*–*
E24 (Vet)	1	508	Chl	N	N (N)	*bla*_TEM_, *bla*_OXA_	–	–	*–*	–	*–*
S25 (Vet)	1	682	AmpNalCip	N	Y (Y)	*bla*_CTX-M_ Grp 9	131	O25:H4	Col156*, IncF RST F1:A-:B20	*bla*_CTX-M-27_	*gad, iha, iss, senB*
		684	AmpTmpTet	Y	– (–)	*–*	58 (CC-155)	O8:H8	Col156*, IncF RST F29:A-:B10, IncX1*, IncY*	*bla*_CTX-M-14_, *bla*_TEM-1B_, *dfrA14, fosA7, QnrS1, tet(A)*	*capU, gad, iroN, iss, lpfA, mchC, senB*
	2	817	AmpNalCip	N	Y (Y)	*bla*_CTX-M_ Grp 9	–	–	*–*	–	*–*
		819	AmpTmpTet	Y	– (–)	*–*	69 (CC-69)	H18	Col(BS512)*, IncQ1	*bla*_TEM-207_, *dfrA7, strA, strB, sul1, sul2, tet(A)*	*air, gad, iss, lpfA*
	3	1026	AmpNalCip	N	Y (Y)	*bla*_CTX-M_ Grp 9	131	O16:H4	Col156*, IncF RST F1:A6:B20	*bla*_CTX-M-27_, *dfrA14*	*espA, espF, gad, iha, iss, nfaE, sat, senB*
		1027	AmpNal	N	Y (Y)	*bla*_CTX-M_ Grp 9	131	O25:H4	Col156*, IncF RST F1:A2:B20	*bla*_CTX-M-27_	*gad, iha, iss, sat, senB*
	4	1233	AmpNalCip	N	Y (Y)	*bla*_CTX-M_ Grp 9, *bla*_AmpC_ (ACC)	131	O25:H12	Col156*, IncF RST F1:A2:B20	*aadA2, bla*_CTX-M-27_, *bla*_TEM-1B_, *dfrA12*	*aaiC, agg3B, gad, iha, iss, nfaE, sat, senB*
		1234	AmpNalCip	N	Y (Y)	*bla*_CTX-M_ Grp 9	–	–	*–*	–	*–*
	5	1242	AmpNalCip	N	Y (Y)	*bla*_CTX-M_ Grp 9	–	–	*–*	–	*–*
		1241	AmpNal	N	Y (Y)	*bla*_CTX-M_ Grp 9	38 (CC-38)	O86:H18	Col(MG828)*, IncB/O/K/Z, IncF RST F51:A-:B10, IncI1 Incomplete ST	*bla*_CTX-M-14_, *dfrA7*	*air, capU, eilA, gad, iha, iss, nfaE, sat, senB*
	6	1322	AmpNalCip	N	Y (Y)	*bla*_CTX-M_ Grp 9	–	–	*–*	–	*–*
		1316	AmpNal	N	Y (Y)	*bla*_CTX-M_ Grp 9	–	–	*–*	–	*–*
S29 (Vet)	5	1034	AmpTmp	N	Y (Y)	*bla*_CTX-M_ Grp 1	–	–	*–*	–	*–*
E31 (Vet)	1	540	None	N	N (N)	*bla*_TEM_, *bla*_OXA_	–	–	*–*	–	*–*
	6	1272	AmpAmc	N	N (Y)	*bla*_AmpC_ (CIT)	–	–	*–*	–	*–*
E41 (Vet)	1	509	AmpAmc	N	N (Y)	*bla*_TEM_, *bla*_OXA_	–	–	*–*	–	*–*
	2	610	AmpAmc	N	N (-)	*bla*_AmpC_ (CIT)	–	–	*–*	–	*–*
E46 (VN)	4	1082	AmpTmpTet	Y	Y (Y)	*bla*_CTX-M_ Grp 1	–	–	*–*	–	*–*
S55 (AS)	1	516	AmpChlTet	Y	N (N)	*bla*_SHV_	–	–	*–*	–	*–*
	6	1133	AmpAmcChlNalTmpTet	Y	– (–)	*–*	75	H8	Col156; IncF RST F29:A-:B10, IncX1;	*fosA7*	*gad, ireA, iroN, iss, lpfA, mchB, mchC, mchF, mcmA, pic, senB*
S59 (Vet)	1	1002	Amp	N	Y (Y)	*bla*_CTX-M_ Grp 9, *bla*_TEM_	127	H31	Col(BS512), Col156*, IncB/O/K/Z, IncF RST F29:A-:B10, IncI1 Incomplete ST	*bla*_CTX-M-14_, *bla*_TEM-1A_	*gad, ireA, iss, senB, vat*
		1005	AmpNalTet	Y	– (–)	*–*	69 (CC-69)	O25:H4	Col(BS512)*, Col156*, IncF RST F95:A-:B1	*bla*_TEM-1B_, *tet(B)*	*air, gad, iss, lpfA, senB*
		1008	AmpNalCip	N	– (–)	*–*	38 (CC-38)	O1:H15	Col(BS512), Col156*, IncF RST F29:A-:B10	*bla*_TEM-1B_	*air, eilA, gad, iss, senB*
	2	1105	Amp	N	Y (Y)	*bla*_CTX-M_ Grp 9, *bla*_TEM_	127	O6:H31	Col156*, IncB/O/K/Z, IncF RST F29:A-:B10, IncI1 Incomplete ST	*bla*_CTX-M-14_, *bla*_TEM-1A_, *QnrS1*	*gad, ireA, iss, senB, vat*
	3	1192	AmpTet	N	Y (Y)	*bla*_CTX-M_ Grp 9, *bla*_TEM_	127	H31	Col(BS512), Col156*, IncB/O/K/Z, IncF RST F29:A-:B10, IncI1 Unknown ST	*aadA2, bla*_CTX-M-14_, *bla*_TEM-1A_, *strB, sul2, tet(A)*	*aaiC, agg3B, cba, gad, iss, senB, vat*
	5	1338	AmpTmpTet	Y	Y (Y)	*bla*_CTX-M_ Grp 9, *bla*_TEM_	Unknown	O16:H19	IncB/O/K/Z, IncF RST F1:A2:B15, IncI1 Incomplete ST	*bla*_CTX-M-27_, *bla*_TEM-1B_, *dfrA8, dfrA14, strA, strB, sul2, tet(B)*	*astA, eae, espA, espI, etpD, iha, iss, nfaE, nleA, nleB, senB, tir*
	6	1345	Amp	N	Y (Y)	*bla*_CTX-M_ Grp 9, *bla*_TEM_	–	–	*–*	–	*–*

*Letter in front of participant’s ID number indicates workplace (E=equine hospital, S=small animal hospital, F=farm animal hospital, U=undergraduate veterinary student).

–, not tested; 3GCR, resistance to at least one third-generation cephalosporin on double disc diffusion testing; Amc, amoxicillin clavulanate resistance; Amp, ampicillin resistance; AS, auxiliary staff; CC, clonal complex; Chl, chloramphenicol resistance; Cip, ciprofloxacin resistance; Grp, group; MDR, multidrug resistance; n, no; Nal, nalidixic acid resistance; ST, sequence type; Tet, tetracycline resistance; UVS, undergraduate veterinary student; Vet, veterinary surgeon; VN, veterinary nurse; Y, yes.

Sixteen participants were found to be carrying resistance genes (*bla*_CTX-M_, *bla*_TEM_, *bla*_SHV_, *bla*_OXA_ and/or *bla*_AmpC_) tested for by PCR assay. PCR detected *bla*_AmpC_ genes in isolates from seven participants; four participants carried *bla*_CIT_ and three *bla*_ACC_. The same resistance genes were detected in multiple samples from six participants; three participants (F03, S25 and S59) were found to be carrying the same resistance genes by PCR assay for multiple weeks and had multiple isolates further characterised by WGS (table 5).

## Discussion

The prevalence of ESBL-producing *E coli* in people working in three veterinary hospitals in the UK was estimated to be 5.95 per cent (95 per cent CI 0.89 to 11.0 per cent). It was hypothesised that a higher carriage rate would be found in this population than in the general population due to contact with domestic animals and antimicrobial use in the veterinary hospital environment. Previous prevalence estimates of asymptomatic control populations in the UK are 1.5 and 7.3 per cent.^5 45^ However, the latter study sampled individuals from an ethnically diverse population.^45^ Such populations may travel internationally to areas with high prevalence of ESBL-producing bacteria, a known risk factor for such carriage.^46 47^ Other recent European studies of healthy asymptomatic volunteers have found prevalence rates varying from 2.3 to 5.8 per cent.^48–51^ Dolejska *et al*^15^ found one (1/12) rectal swab from equine veterinary clinic staff positive for ESBL-producing *E coli* in the Czech Republic. This study identified one positive faecal swab from the EH (n=20), one from the FAH (n=32) and three from the SAH (n=32). Of the five samples from the cross-sectional study containing ESBL-producing *E coli*, the three carrying *bla*_CTX-M-15_ were collected from participants working in each hospital, and the two samples carrying *bla*_CTX-M-14_ and *bla*_CTX-M-27_ were isolated from participants from the SAH. Interestingly, *bla*_CTX-M-14_ is commonly associated with livestock, yet was isolated from a participant in the SAH.^52^ Isolates from two of the participants carrying *bla*_CTX-M-15_ were identified as ST405, which is globally associated with CTX-M-15.^53^ Isolate 350, belonging to serotype O25:H4 and ST131, was found to carry *bla*_CTX-M-27_, which is increasingly associated with the human pandemic *E coli* clone O25:H4-ST131.^54^

High prevalences of AMR (60.7 per cent; 95 per cent CI 50.3 to 71.2 per cent), MDR (32.1 per cent; 95 per cent CI 22.2 to 42.1 per cent) and ciprofloxacin-resistant (11.9 per cent; 95 per cent CI 4.98 to 18.8 per cent) *E coli* were found in cross-sectional study participants. The fluoroquinolone ciprofloxacin is designated a highest-priority critically important antimicrobial, which should not be used as a first-line treatment in people and animals,^1^ therefore this high prevalence of ciprofloxacin resistance was unexpected. It is well known that people occupationally exposed to animals have higher rates of carriage of MRSA than the general population.^20^ Additionally, studies investigating the carriage of AMR and ESBL-producing bacteria by people working with animals have found higher carriage rates in farmers than the general population.^12–14 55^ Notably, Huijbers *et al*^14 55^ reported the prevalence of ESBL-producing and AmpC-producing *E coli* in broiler farmers to be 19.1 per cent compared with 5.1 per cent in the local surrounding non-farming community.

High rates of carriage of AMR and ESBL-producing bacteria have also been found in the populations of hospitalised domestic species in the hospitals investigated in this study. In the SAH, 31.6 per cent of hospitalised cats and dogs were found to be carrying MDR bacteria and 26.5 per cent carried ESBL-producing bacteria.^10^ Maddox *et al*^17^ found 47.7 per cent of samples from horses hospitalised in the EH carried MDR *E coli* and 53.4 per cent carried ESBL-producing *E coli*. Following hospitalisation, equine faecal *E coli* AMR profiles significantly altered and carriage of AMR and MDR *E coli* was significantly higher. Within the hospital environment, patients’ commensal flora are exposed to greater selection pressures for AMR than when the patient is in the community. These selection pressures are influenced by the widespread use of antimicrobials and disinfectants and co-habitation with other patients being treated with antimicrobials or carrying AMR bacteria.^16 17^ The high levels of carriage of AMR *E coli* in the human and animal populations within the study hospitals may be due to transmission from hospitalised animals or the hospital environment, acquisition of resistance determinants from other bacteria from these sources or from the individual’s gut flora, or an increase in resistant *E coli* already present in the gastrointestinal flora.^17^

This study is the first to longitudinally study the faecal carriage of AMR and ESBL-producing *E coli* by people working in veterinary hospitals. Interestingly, longitudinal sampling revealed that a much higher percentage of participants (7/27; 25.9 per cent (95 per cent CI 9.40 to 42.5 per cent)) carried ESBL-producing *E coli* at least once during a six-week study period than during cross-sectional sampling, with persistent carriage of ESBL-producing *E coli* in at least five out of six samples from three participants. Zurfluh *et al*^56^ demonstrated persistent carriage in an international traveller to India of <8 months duration. Moreover, two longitudinal Swedish studies have investigated carriage of ESBL-producing *E coli* by international travellers and both detected persistent colonisation of participants of up to three years duration.^46 47^ Tham *et al*^47^ demonstrated that ESBL-producing *E coli* strains isolated from some patients changed over the course of the longitudinal study. In this study, ESBL-producing isolates with identical resistance profiles with the same resistance genes and MLST type were isolated from consecutive weekly samples. However, ESBL-producing *E coli* isolates were found with diverse resistance profiles and varying resistance genes from individual samples and participants and ESBL-producing isolates with the same MLST type were isolated from individual participants in non-consecutive weeks. These findings could be explained by the transfer of resistance genes, for example, by plasmid transfer, from a colonising *E coli* strain to the resident commensal *E coli* in the participant’s gut flora. It is possible that transmission of resistance genes and/or isolates is occurring between the human and animal populations within these hospitals. Transmission events have been previously demonstrated in veterinary environments between people and animals^15 19^ and future studies should aim to concurrently sample the hospital patients, staff and environment to investigate transmission. It is also possible that, during the study, participants were colonised by multiple ESBL-producing *E coli*, but not all were cultured from each sample due to variations in faecal shedding or limitations in microbiological culture. Additionally, participants may have become colonised with a different ESBL-producing *E coli* during the study. Ultimately, this study has shown that people working in veterinary environments may carry ESBL-producing *E coli* transiently for a single week or more persistently over multiple weeks. To enable comparisons, further work should include community-based longitudinal cohort studies.

Multivariable logistic regression analysis revealed that workplace, direct contact with animal faeces at work and hospitalisation in the last six months were independently associated with carriage of trimethoprim-resistance *E coli* (P<0.001). Previous studies have identified recent antimicrobial treatment and hospitalisation,^57^ and contact with domestic animals,^58^ horses^55^ and broiler chickens^14 59^ as risk factors for carriage of AMR and ESBL-producing *Enterobacteriaceae.* However, regression analyses could not construct any other significant (P<0.05) multivariable models for other resistance outcomes. It was difficult to draw statistically valid conclusions from many of the univariable and multivariable logistic regression models due to difficulties with participant recruitment. This resulted in similarity in some questionnaire responses and a low observed prevalence of some resistance outcomes.

While the use of convenience sampling to select participating hospitals and study participants may have introduced some bias, it is unlikely that this would affect the observed prevalence of faecal carriage of AMR or ESBL-producing *E coli*. Poor compliance for faecal sample submission for health screening and research studies is well documented worldwide.^5^ Reasons cited for non-submission often include procrastination, inconvenience and perceived unpleasantness of collecting a faecal sample. To improve compliance in this study, participants were asked to provide a swab of faeces as opposed to a whole faecal sample. Advertisement of the study was frequent and ubiquitous and sample packs were conveniently located to increase participation. Despite this, participant recruitment was a difficult process. Moreover, both cross-sectional and longitudinal study participants were overwhelmingly qualified veterinary surgeons. This may be because the study hospitals are teaching and referral hospitals where veterinary surgeons are likely to be involved in veterinary research. Thus, these individuals may be more motivated to participate in research studies, despite having to provide a faecal swab, than those members of staff and students not actively involved in research. Sampling techniques other than convenience sampling would not have been effective in this study due to the reluctance of the study population to provide faecal swabs for analysis. While the required sample size of 65 was achieved in this study, more work needs to be done to improve the perception of human faecal sampling to ensure that research studies not able to offer compensation in return for sampling achieve sufficient sample sizes.

In conclusion, this study is the first to estimate the prevalence of ESBL-producing *E coli* in people working in three veterinary hospitals in the UK (5.95 per cent; 95 per cent CI 0.89 to 11.0 per cent). It is the first longitudinal study of faecal carriage of AMR and ESBL-producing *E coli* by people working in veterinary hospitals and found that 25.9 per cent of longitudinal participants provided at least one ESBL-producing *E coli*-positive sample during the six-week study period. This study demonstrates that people working in veterinary environments are carriers of ESBL-producing *E coli* and may act as a reservoir of ESBL-producing bacteria in the community. Prior intestinal colonisation with ESBL-producing bacteria is a risk factor for nosocomial infection and carriers may be a source of ESBL-producing bacteria in the population.^6 7^ This has serious implications for carriers themselves, their families and their communities. It is vital that veterinary hospitals implement gold-standard biosecurity to prevent transmission of MDR and ESBL-producing bacteria between patients and staff. Healthcare providers should be made aware that people working in veterinary hospitals are a high-risk population for carriage of MDR and ESBL-producing bacteria and that this poses a risk to the carrier and for transmission of resistance throughout the wider community.
