# Supplementary material for: Prevalence of faecal carriage of extended-spectrum β-lactamase (ESBL)-producing Escherichia coli in veterinary hospital staff and students
Source: Vet Rec Open. 2019 Jan 7;6(1):e000307. doi: 10.1136/vetreco-2018-000307 (PMC6327872; doi:10.1136/vetreco-2018-000307)
Supplement: Supplementary data [file vetreco-2018-000307supp001.pdf]

UNIVERSITY OF LIVERPOOL

Ref | | | |

Questionnaire

Working in Veterinary Environments and the Risk of Acquiring Antibiotic-Resistant Bacteria

As part of the research into the potential role domestic animals play in the spread of antibiotic resistance we are asking people who work with companion animals, horses and livestock to provide us with a faecal sample swab and to fill in this questionnaire.

Your participation in this study will help us to better understand the role domestic animals play in the spread of antibiotic resistance through direct contact with people.

Completion and return of this questionnaire alongside provision of a faecal sample swab will be taken as informed consent for the use of the sample and data in our study as described in the accompanying letter.

Participation in this study is entirely voluntary and all responses will remain completely confidential.

All information from this questionnaire is strictly confidential and will be available only to the investigators. All data is anonymised and no individuals will be identifiable from published data.

Answering the questions

This questionnaire consists of the three short sections:

Section one: Information about you

Section two: Information about your work

Section three: Information about your home environment

Please answer all questions by marking a ☒ in the box or by writing in the boxes provided using BLOCK CAPITAL letters.

E.g. Have you received antibiotic treatment within the last 6 months?

☒ Yes ☐ No ☐ Don't know

If you make a mistake or change your answer please fill in the first box completely and put a clear cross in the correct box.

Once completed please place the questionnaire and your faecal sample swab in the envelope provided and return to the collection box located in your workplace.

If you have any questions or queries please contact:

Miss Alexandra Royden
Department of Epidemiology and Population Health
University of Liverpool
Neston
CH64 7TE
0151 794 6079
A.L.Royden@liverpool.ac.uk

ABOUT YOU

1. How old are you?

   ☐ 16-25 years ☐ 26-35 years

   ☐ 36-45 years ☐ 46-55 years

   ☐ 56-65 years ☐ Over 65 years

2. Have you received any antibiotic treatment within the last 6 months?

   ☐ Yes ☐ No ☐ Don't know

   If yes, when was the most recent treatment course?

   ☐ Within last month

   ☐ 2-3 months ago

   ☐ Over 3 months ago

   Please indicate antibiotic treatment (e.g. penicillin), if known:

3. Have you been hospitalised within the last 6 months?

   ☐ Yes ☐ No ☐ Don't know

   If yes, how long ago?

   ☐ Within last month

   ☐ 2-3 months ago

   ☐ Over 3 months ago

4. When did you last travel outside the UK?

   ☐ Within last 3 months ☐ 10-12 months ago

   ☐ 4-6 months ago ☐ Over 12 months ago

   ☐ 7-9 months ago

   Please indicate where you travelled to on this occasion:

ABOUT YOUR WORK

5. What is your role?

☐ Veterinary Surgeon ☐ Veterinary Student

☐ Veterinary Nurse ☐ Veterinary Nursing Student

☐ Administrative Role ☐ Volunteer

☐ Animal Care Assistant/Yard Staff/Animal Husbandry Role or Similar

6. How long have you worked here? If you are a veterinary student, please move on to Question 8.

☐☐ Years ☐☐ Months

7. Do you work here full-time?

☐ Yes ☐ No ☐ Prefer not to say

8. Which animals do you come into direct contact with <u>in this workplace</u> on a daily basis? (Tick any boxes which apply)

☐ Dogs ☐ Cats

☐ Horses ☐ Farm animals (please specify)_____________________

☐ Other (please specify)____

9. Apart from animals you have regular contact with in this workplace, have you had contact with any other animals in a work capacity in the last three months? For example, veterinary students undertaking placements or previous rotations. (Tick any boxes which apply)

☐ None ☐ Dogs ☐ Cats

☐ Horses ☐ Farm animals (please specify)_____________________

☐ Other (please specify)____

10. Please specify the percentage of your time at work spent on the following:

Direct contact with animals ☐ Cleaning kennels, stables, pens, etc. ☐ Office work ☐

Other (please specify)

11. Do you have direct contact with animal faeces at work?

☐ Yes ☐ No

ABOUT YOUR HOME ENVIRONMENT

12. Has any member of your household been hospitalised within the last 6 months?

☐ Yes ☐ No ☐ Don't know

13. Do you have direct contact with companion animals or horses at home?

☐ Yes ☐ No

If yes, which animals do you come into contact with at home? (Tick any boxes which apply)

☐ Dogs ☐ Horses

☐ Cats ☐ Small mammals (e.g. hamster)

☐ Other (please specify) ______________________________

14. Do you have direct contact with farm animals at home?

☐ Yes ☐ No

If yes, which farm animals do you come into contact with at home? (Tick any boxes which apply)

☐ Dairy cattle ☐ Pigs

☐ Beef cattle ☐ Poultry

☐ Sheep ☐ Other (please specify)______________________________

15. Do you eat meat?

☐ Yes ☐ No

If yes, please indicate which meat you eat three or more times weekly. (Tick any boxes which apply)

☐ Beef ☐ Poultry

☐ Lamb ☐ Fish

☐ Pork ☐ Other (please specify)____________________________

16. Do you eat salad leaves three or more times weekly?

☐ Yes ☐ No

We would also like to follow individuals longitudinally to look at variation in shedding of antibiotic-resistant bacteria over time. This would involve providing additional faecal sample swabs. This is entirely voluntary and there is no obligation to participate if you are contacted. If you are willing to take part in this further study, please indicate below and provide an email address.

17. I would be happy for the study team to contact me

☐ Yes ☐No

18. My email address is ______________________________

We welcome feedback from our participants. Please indicate any further comments or concerns regarding this study:

If you would like to discuss your comments further, please do not hesitate to email the study team at A.L.Royden@liverpool.ac.uk.

Many thanks for your participation
