# Supplementary material for: Prevalence of faecal carriage of extended-spectrum β-lactamase (ESBL)-producing Escherichia coli in veterinary hospital staff and students
Source: Vet Rec Open. 2019 Jan 7;6(1):e000307. doi: 10.1136/vetreco-2018-000307 (PMC6327872; doi:10.1136/vetreco-2018-000307)
Supplement: Supplementary data [file vetreco-2018-000307supp002.docx]

**Supplementary Material**

Table 1: Resistance profiles of 82 unique AMR faecal *E. coli* isolates from 51 (N=84) samples. Amp = ampicillin resistance; Amc = amoxicillin-clavulanate resistance; Nal = nalidixic acid resistance; Cip = ciprofloxacin resistance; Chl = chloramphenicol resistance; Tmp = trimethoprim resistance; Tet = tetracycline resistance.

| Resistance Profile | Total Number of Isolates (%) | Farm Animal Hospital | Equine Hospital | Small Animal Hospital |
| --- | --- | --- | --- | --- |
| Amp | 11 (13.4) | 2 | 4 | 5 |
| AmpTmpTet | 7 (8.54) | 5 | 0 | 2 |
| AmpTmp | 6 (7.32) | 3 | 2 | 1 |
| AmpTet | 5 (6.11) | 2 | 1 | 2 |
| Tet | 5 (6.11) | 1 | 2 | 2 |
| AmpChlNalCipTmpTet | 4 (4.88) | 2 | 1 | 1 |
| AmpAmcTet | 4 (4.88) | 1 | 1 | 2 |
| AmpNalTet | 4 (4.88) | 1 | 2 | 1 |
| AmpNalCipTmpTet | 3 (3.66) | 0 | 1 | 2 |
| Nal | 3 (3.66) | 2 | 0 | 1 |
| TmpTet | 3 (3.66) | 2 | 1 | 0 |
| AmpAmc | 2 (2.43) | 0 | 0 | 2 |
| AmpAmcChlNalCipTmp | 2 (2.43) | 1 | 1 | 0 |
| AmpAmcChlNalCipTmpTet | 2 (2.43) | 1 | 0 | 1 |
| AmpAmcNalCip | 2 (2.43) | 1 | 1 | 0 |
| AmpAmcTmpTet | 2 (2.43) | 0 | 1 | 1 |
| AmpNal | 2 (2.43) | 1 | 0 | 1 |
| AmpNalCip | 2 (2.43) | 1 | 0 | 1 |
| AmpNalTmpTet | 2 (2.43) | 0 | 1 | 1 |
| Tmp | 2 (2.43) | 2 | 0 | 0 |
| AmpAmcChlNalTmpTet | 1 (1.22) | 1 | 0 | 0 |
| AmpAmcNalTmpTet | 1 (1.22) | 1 | 0 | 0 |
| AmpAmcTet | 1 (1.22) | 1 | 0 | 0 |
| AmpAmcTmpTet | 1 (1.22) | 1 | 0 | 0 |
| AmpNalCipTet | 1 (1.22) | 1 | 0 | 0 |
| AmcTet | 1 (1.22) | 1 | 0 | 0 |
| Chl | 1 (1.22) | 0 | 0 | 1 |
| ChlNalCipTmp | 1 (1.22) | 1 | 0 | 0 |
| NalTmpTet | 1 (1.22) | 0 | 1 | 0 |
| TOTAL | 82 | 35 | 20 | 27 |

Table 2: Contig Size and Total Genome Length of 53 Sequenced Isolates from 84 Cross-Sectional Study Participants and 27 Longitudinal Study Participants

| **Isolate** | **SRA Accession No.** | **# contigs (>= 0 bp)** | **# contigs (>= 1000 bp)** | **Total length (>= 0 bp)** | **Total length (>= 1000 bp)** | **# contigs** | **Largest contig** | **Total length** | **GC (%)** | **N50** | **N75** | **L50** | **L75** | **# N's per 100 kbp** |
| --- | --- | --- | --- | --- | --- | --- | --- | --- | --- | --- | --- | --- | --- | --- |
| 5 | SRR7211987 | 1,859 | 342 | 5,872,594 | 5,024,440 | 1,155 | 187,621 | 5,548,908 | 50.66 | 57,151 | 21,127 | 29 | 69 | 0.00 |
| 12 | SRR7211950 | 334 | 249 | 5,362,995 | 5,316,593 | 293 | 205,298 | 5,347,280 | 50.53 | 48,818 | 24,507 | 34 | 73 | 0.00 |
| 17 | SRR7211976 | 284 | 218 | 5,396,051 | 5,361,400 | 253 | 230,032 | 5,384,921 | 50.53 | 59,074 | 28,372 | 29 | 62 | 0.00 |
| 36 | SRR7211971 | 380 | 285 | 5,156,262 | 5,101,194 | 340 | 101,167 | 5,140,607 | 50.75 | 36,147 | 19,259 | 48 | 97 | 0.00 |
| 59 | SRR7211981 | 2,302 | 776 | 6,698,927 | 5,811,405 | 1,754 | 95,020 | 6,454,061 | 50.81 | 14,848 | 4,423 | 112 | 302 | 0.00 |
| 73 | SRR7211984 | 1,113 | 379 | 5,321,909 | 4,921,950 | 734 | 113,129 | 5,149,608 | 50.83 | 24,771 | 11,472 | 63 | 139 | 0.00 |
| 91 | SRR7211958 | 237 | 177 | 4,600,265 | 4,571,271 | 200 | 130,830 | 4,587,710 | 50.74 | 57,728 | 29,291 | 28 | 56 | 0.00 |
| 101 | SRR7211966 | 255 | 198 | 4,869,769 | 4,840,117 | 224 | 292,025 | 4,859,424 | 50.56 | 59,160 | 29,687 | 27 | 55 | 0.00 |
| 121 | SRR7211951 | 1,002 | 809 | 5,022,063 | 4,900,445 | 946 | 42,245 | 4,997,170 | 50.98 | 8,838 | 4,810 | 170 | 358 | 0.00 |
| 123 | SRR7211953 | 3,220 | 1,604 | 4,225,000 | 3,152,798 | 2,904 | 44,464 | 4,079,028 | 51.53 | 1,703 | 1,043 | 743 | 1,513 | 0.00 |
| 131 | SRR7211980 | 338 | 195 | 5,150,770 | 5,082,915 | 247 | 197,963 | 5,116,253 | 57.73 | 53,341 | 27,525 | 31 | 63 | 0.00 |
| 151 | SRR7211974 | 368 | 271 | 5,402,980 | 5,345,782 | 334 | 156,520 | 5,389,888 | 50.62 | 45,027 | 23,451 | 38 | 80 | 0.00 |
| 161 | SRR7211973 | 417 | 328 | 5,215,801 | 5,161,023 | 385 | 131,533 | 5,202,452 | 50.92 | 31,869 | 15,674 | 49 | 109 | 0.00 |
| 175 | SRR7211975 | 575 | 408 | 5,464,039 | 5,365,303 | 516 | 126,999 | 5,441,592 | 50.69 | 31,302 | 12,258 | 48 | 118 | 0.00 |
| 181 | SRR7211986 | 354 | 248 | 5,369,570 | 5,310,407 | 309 | 137,759 | 5,353,002 | 50.71 | 44,391 | 24,904 | 37 | 77 | 0.00 |
| 199 | SRR7211985 | 279 | 214 | 5,131,764 | 5,092,163 | 258 | 170,989 | 5,123,345 | 50.45 | 57,245 | 27,896 | 27 | 60 | 0.00 |
| 200 | SRR7211945 | 335 | 254 | 5,184,487 | 5,137,861 | 301 | 182,231 | 5,172,605 | 50.77 | 36,924 | 20,135 | 41 | 88 | 0.00 |
| 229 | SRR7211946 | 306 | 224 | 5,159,369 | 5,108,464 | 280 | 169,519 | 5,149,064 | 50.80 | 49,204 | 24,348 | 33 | 70 | 0.00 |
| 254 | SRR7211943 | 230 | 171 | 4,824,929 | 4,792,553 | 204 | 157,974 | 4,815,075 | 50.66 | 54,410 | 30,036 | 26 | 56 | 0.00 |
| 258 | SRR7211944 | 172 | 117 | 4,856,132 | 4,829,153 | 139 | 261,815 | 4,844,011 | 50.57 | 96,214 | 48,816 | 17 | 35 | 0.00 |
| 266 | SRR7211941 | 269 | 209 | 4,776,821 | 4,745,278 | 240 | 139,515 | 4,766,776 | 50.82 | 40,364 | 23,197 | 37 | 76 | 0.00 |
| 267 | SRR7211942 | 343 | 252 | 4,740,038 | 4,685,927 | 310 | 89,979 | 4,727,181 | 50.99 | 34,568 | 17,929 | 45 | 93 | 0.00 |
| 288 | SRR7211967 | 563 | 234 | 5,457,260 | 5,300,160 | 347 | 146,540 | 5,375,678 | 50.59 | 50,247 | 23,266 | 32 | 71 | 0.00 |
| 299 | SRR7211968 | 2,949 | 479 | 6,930,357 | 5,557,437 | 1,806 | 213,992 | 6,405,861 | 50.86 | 34,084 | 8,501 | 49 | 134 | 0.02 |
| 331 | SRR7211935 | 319 | 201 | 5,115,662 | 5,048,333 | 271 | 157,044 | 5,096,533 | 50.76 | 50,597 | 29,164 | 32 | 65 | 0.00 |
| 350 | SRR7211938 | 206 | 149 | 5,057,670 | 5,025,581 | 176 | 304,708 | 5,044,531 | 50.74 | 76,090 | 39,072 | 17 | 39 | 0.00 |
| 380 | SRR7211970 | 543 | 314 | 4,939,904 | 4,812,401 | 438 | 101,203 | 4,893,381 | 51.00 | 32,655 | 15,233 | 50 | 105 | 0.00 |
| 386 | SRR7211982 | 739 | 523 | 5,336,412 | 5,214,466 | 651 | 83,708 | 5,306,986 | 50.98 | 17,765 | 9,296 | 93 | 198 | 0.00 |
| 411 | SRR7211969 | 3,370 | 1,654 | 4,388,290 | 3,256,873 | 3,003 | 10,437 | 4,218,870 | 51.09 | 1,691 | 1,053 | 770 | 1,564 | 0.07 |
| 682 | SRR7211955 | 418 | 334 | 4,988,821 | 4,938,181 | 390 | 104,859 | 4,977,756 | 50.95 | 25,002 | 13,517 | 56 | 124 | 0.00 |
| 684 | SRR7211972 | 2,089 | 596 | 6,518,704 | 5,667,012 | 1,477 | 152,051 | 6,241,210 | 50.72 | 28,045 | 7,039 | 57 | 157 | 0.02 |
| 736 | SRR7211983 | 321 | 234 | 4,969,643 | 4,915,351 | 290 | 129,271 | 4,956,788 | 50.74 | 42,581 | 23,905 | 35 | 72 | 0.00 |
| 770 | SRR7211936 | 2,854 | 815 | 6,643,111 | 5,507,475 | 1,952 | 57,731 | 6,247,061 | 51.02 | 12,994 | 3,465 | 127 | 340 | 0.03 |
| 819 | SRR7211937 | 990 | 735 | 4,787,933 | 4,626,954 | 923 | 45,077 | 4,757,783 | 51.29 | 9,185 | 5,196 | 161 | 333 | 0.00 |
| 1002 | SRR7211963 | 347 | 215 | 5,188,523 | 5,115,751 | 285 | 218,094 | 5,161,338 | 50.64 | 49,713 | 27,845 | 34 | 70 | 0.00 |
| 1005 | SRR7211964 | 349 | 248 | 5,252,706 | 5,196,034 | 302 | 152,471 | 5,235,435 | 50.76 | 47,945 | 22,489 | 33 | 74 | 0.00 |
| 1008 | SRR7211965 | 300 | 238 | 5,308,517 | 5,272,626 | 273 | 121,114 | 5,298,959 | 50.46 | 45,552 | 24,644 | 37 | 78 | 0.00 |
| 1011 | SRR7211959 | 2,131 | 1,056 | 6,634,106 | 5,958,394 | 1,945 | 63,576 | 6,566,092 | 50.69 | 8,843 | 3,250 | 180 | 492 | 0.00 |
| 1012 | SRR7211960 | 303 | 237 | 4,973,996 | 4,936,006 | 275 | 130,087 | 4,963,031 | 50.82 | 42,191 | 24,307 | 36 | 73 | 0.00 |
| 1017 | SRR7211961 | 1,587 | 657 | 6,933,843 | 6,374,425 | 1,285 | 204,009 | 6,796,451 | 50.54 | 33,390 | 7,302 | 50 | 145 | 0.03 |
| 1026 | SRR7211962 | 1,058 | 233 | 5,574,889 | 5,142,189 | 644 | 234,647 | 5,401,984 | 50.75 | 73,130 | 29,463 | 22 | 51 | 0.00 |
| 1027 | SRR7211956 | 332 | 249 | 5,035,181 | 4,988,244 | 301 | 124,417 | 5,023,140 | 50.88 | 38,579 | 18,955 | 38 | 84 | 0.00 |
| 1105 | SRR7211957 | 1,891 | 395 | 6,346,448 | 5,498,169 | 1,285 | 220,086 | 6,070,634 | 50.58 | 53,781 | 16,957 | 30 | 77 | 0.00 |
| 1133 | SRR7211947 | 256 | 207 | 5,100,948 | 5,071,465 | 238 | 183,953 | 5,093,423 | 50.53 | 46,983 | 25,504 | 31 | 68 | 0.00 |
| 1151 | SRR7211948 | 507 | 369 | 5,098,870 | 5,022,378 | 446 | 91,925 | 5,075,520 | 50.99 | 27,785 | 14,347 | 55 | 119 | 0.02 |
| 1192 | SRR7211949 | 2,611 | 609 | 6,541,127 | 5,433,005 | 1,672 | 87,534 | 6,110,802 | 50.94 | 18,933 | 5,826 | 89 | 224 | 0.02 |
| 1214 | SRR7211952 | 1,329 | 388 | 5,965,158 | 5,436,293 | 911 | 105,394 | 5,776,909 | 50.62 | 33,637 | 14,309 | 54 | 120 | 0.00 |
| 1233 | SRR7211954 | 1,716 | 369 | 6,065,650 | 5,290,308 | 1,164 | 191,062 | 5,813,366 | 50.81 | 60,184 | 18,598 | 28 | 69 | 0.03 |
| 1241 | SRR7211939 | 331 | 237 | 5,378,148 | 5,321,744 | 298 | 143,885 | 5,364,906 | 50.60 | 56,813 | 28,032 | 30 | 61 | 0.00 |
| 1279 | SRR7211940 | 311 | 191 | 5,442,345 | 5,377,694 | 255 | 288,702 | 5,421,260 | 50.58 | 86,835 | 40,994 | 20 | 43 | 0.00 |
| 1281 | SRR7211978 | 365 | 261 | 5,247,352 | 5,189,076 | 322 | 208,511 | 5,231,590 | 50.78 | 43,995 | 22,328 | 35 | 79 | 0.02 |
| 1286 | SRR7211977 | 215 | 167 | 4,989,103 | 4,958,833 | 200 | 297,336 | 4,982,839 | 50.81 | 86,956 | 40,317 | 18 | 40 | 0.00 |
| 1338 | SRR7211979 | 4,550 | 1,986 | 8,287,050 | 6,659,712 | 3,983 | 33,061 | 8,044,173 | 51.08 | 3,699 | 1,394 | 561 | 1,455 | 0.04 |
